# Carotenoids from Marine Microalgae as Antimelanoma Agents

**DOI:** 10.3390/md20100618

**Published:** 2022-09-29

**Authors:** Christiane Adrielly Alves Ferraz, Raphaël Grougnet, Elodie Nicolau, Laurent Picot, Raimundo Gonçalves de Oliveira Junior

**Affiliations:** 1 UMR 8038 CiTCoM, Faculté de Santé, UFR Pharmacie, Université Paris Cité, 75006 Paris, France; 2 Laboratoire PHYTOX/GENALG, IFREMER, 44311 Nantes, France; 3 UMR CNRS 7266 LIENSs, La Rochelle Université, 17042 La Rochelle, France

**Keywords:** marine carotenoids, marine pigments, melanoma, pigments, skin cancer

## Abstract

Melanoma cells are highly invasive and metastatic tumor cells and commonly express molecular alterations that contribute to multidrug resistance (e.g., BRAF^V600E^ mutation). Conventional treatment is not effective in a long term, requiring an exhaustive search for new alternatives. Recently, carotenoids from microalgae have been investigated as adjuvant in antimelanoma therapy due to their safety and acceptable clinical tolerability. Many of them are currently used as food supplements. In this review, we have compiled several studies that show microalgal carotenoids inhibit cell proliferation, cell migration and invasion, as well as induced cell cycle arrest and apoptosis in various melanoma cell lines. MAPK and NF-ĸB pathway, MMP and apoptotic factors are frequently affected after exposure to microalgal carotenoids. Fucoxanthin, astaxanthin and zeaxanthin are the main carotenoids investigated, in both in vitro and in vivo experimental models. Preclinical data indicate these compounds exhibit direct antimelanoma effect but are also capable of restoring melanoma cells sensitivity to conventional chemotherapy (e.g., vemurafenib and dacarbazine).

## 1. Introduction

Marine organisms represent an exceptional source of bioactive compounds, which a large part correspond to allelopathic molecules with cytotoxicity and/or antiproliferative activity against cancer cells. Since pioneering work in marine pharmacology in the 1950s–1970s, thousands of molecules with novel chemical structures and mechanisms of action have been discovered in marine organisms [1]. The potential for discovery of novel molecules in the coming decades remains significant, particularly for the development of anticancer drugs [2].

A considerable number of studies in marine pharmacology focus on algae compounds. Algae are eukaryotic organisms lacking a stem, root, leaves or flower, able to perform oxygenic photosynthesis and typically living in an aquatic (marine or freshwater) environment [3]. The health applications of micro- and macroalgae are very numerous and concern various fields: food, nutrition/health, prevention and treatment of diet-related diseases (malnutrition, obesity, diabetes, cancers, caries), prevention and treatment of age-related diseases (neurodegeneration, AMD, cardiac problems, tissue engineering), production of innovative drugs (anti-venoms, anticoagulants, antithrombotics, anticancer, antivirals, cytokines, hormones, vaccines, etc.). It is estimated that there are about 1500 species of green macroalgae, 1800 of brown macroalgae and 6500 of red macroalgae. Microalgae, on the other hand, are estimated to comprise 30,000 to 1,000,000 green, red, and brown species (depending on the source) grouped into 12 distinct phyla [4]. This definition of algae excludes cyanobacteria (prokaryotes), which are also the focus of much work to identify molecules of pharmaceutical interest.

Although they have been less studied for pharmaceutical applications, microalgae present an exceptional potential because of the possibility to produce them in large quantities under standardized conditions, and their metabolic plasticity allowing the biosynthesis and biorefining of a wide range of molecules, including polyphenols, pigments, fatty acids/triglycerides ceramides, oxylipins, heterocycles, vitamins and minerals [5]. Recent studies have pointed to microalgae as a source of new anticancer compounds, especially carotenoids. Fucoxanthin, astaxanthin, and zeaxanthin are some examples of microalgae carotenoids capable not only of inhibiting tumor cell proliferation, but also of preventing cell migration and invasion, and inducing apoptosis and cell cycle arrest in different cancer cell lines [6,7,8,9]. When combined with conventional anticancer drugs, these carotenoids act as chemosensitizers, preventing multidrug resistance mechanisms and restoring the sensitivity of tumor cells to chemotherapy [9,10]. In this review, we focus on describing the potential of marine carotenoids specifically on cutaneous melanoma. Below, we have gathered data on melanoma, its pathogenicity and recent treatments, as well as the use of carotenoids purified from microalgae as antimelanoma agents or in association with conventional chemotherapy.

## 2. Melanoma and Multidrug Resistance

Since 2005, the World Health Organization (WHO) has identified cutaneous melanoma as a major priority public health problem. Although it accounts for less than 2% of all skin cancers, it is the most aggressive form responsible for 90% of skin cancer deaths [11]. Approximately 232,000 new cases (1.7% of all malignancies) and 55,500 deaths (0.7% of total cancer mortality) occur worldwide each year, bringing the global average incidence to 10 cases per 100,000 population. In Europe, more than 20,000 people die from melanoma each year, and melanoma is the most common tumor in young adults aged 25–35 years [12].

In addition to phenotypic factors (e.g., skin, hair and eye color), exposure to ultraviolet radiation, the presence of nevi, and family history are the major risk factors associated with the development of melanoma [13]. Its aggressive nature is mainly due to genomic alterations and their post-transcriptional consequences. Overall, melanoma cells express proliferative signaling pathways that are often activated even in the absence of growth factors (e.g., the RAS/RAF/MEK pathway) [14], very readily develop escape mechanisms to apoptosis, stimulate neo-angiogenesis, induce the expression of immunosuppressive factors, and inhibit key immune checkpoints [15].

### 2.1. Molecular Mechanisms Involved in Melanoma Progression

Melanoma is one of the most genetically and clinically heterogeneous cancers due to the complex molecular mechanisms involved in its progression. However, deregulation of cell cycle control, alterations in the RAS/RAF/MEK and AKT/PI3K pathways, and escape from the immune response are notable features of all metastatic melanomas.

#### 2.1.1. Cell Cycle Deregulation

**Figure 1:**
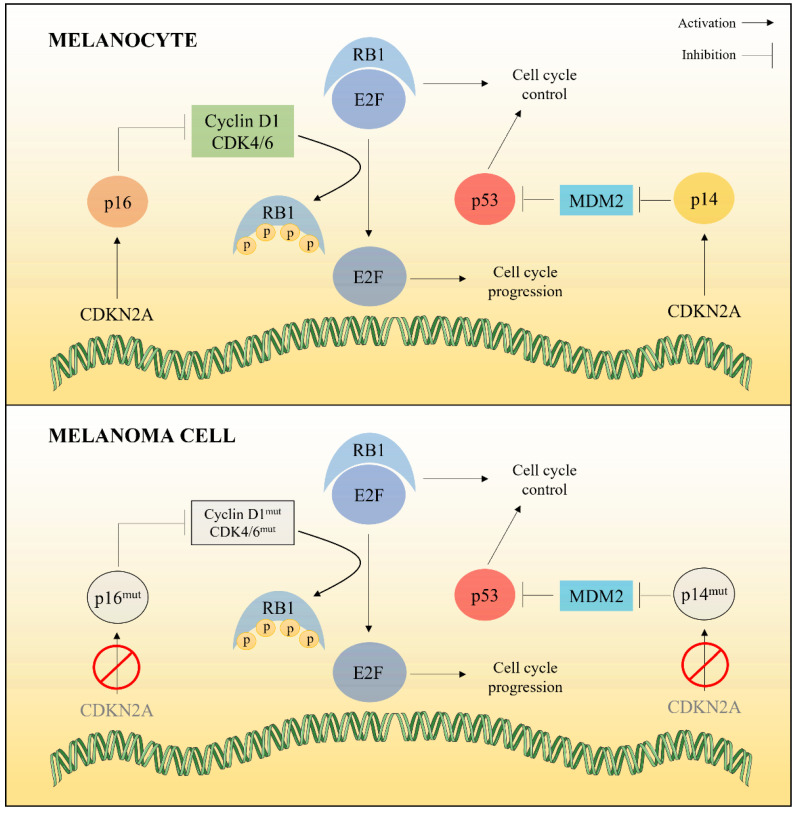
Major signaling pathways involved in cell cycle regulation and their respective alterations in melanoma cells. CDKN2A gene is often mutated in melanoma cells, resulting in decreased expression of cell cycle regulatory proteins (p14 and p16). These proteins can also be mutated, as well as cyclins and CDKs, leading to cell cycle progression.

Cell cycle dysregulation in melanoma cells is responsible for uncontrolled cell proliferation. The CDKN2A (Cyclin-Dependent Kinase Inhibitor 2A) gene mutation, present in 25–50% of familial melanoma cases, has been associated with an elevated risk of melanoma. This gene encodes two distinct proteins, p16/^INK4A^ and p14/^ARF^, which act as tumor suppressors through the negative regulation of pathways involving Rb1 (retinoblastoma protein 1) and p53, respectively [16]. In its unphosphorylated form, Rb1 scavenges the transcription factor E2F in the cytoplasm, thereby blocking the expression of genes essential for cell cycle progression from G1 to S phase. Phosphorylation of Rb1 leads to the release of E2F and the expression of genes responsible for cell cycle progression. This phosphorylation is mediated by a catalytic complex composed of cyclin D1 and CDK4 or CDK6, whose activity depends on p16/^INK4A^ levels. The cyclin D1-CDK4/6 complex is inhibited in the presence of p16/INK4A, which reduces Rb1 phosphorylation and induces cell cycle arrest in G1/S phase (Figure 1). In melanoma cells, genetic mutations affecting the CDKN2A locus cause suppression of p16/^INK4A^, resulting in uncontrolled cell cycle progression. Mutations in the CDK4 gene can also occur, leading to its overexpression and thus constitutive activation of the cyclin D1-CDK4 complex [17,18].

Inhibition of the p53 protein also plays a major role in the development of melanoma. p53 activity is controlled by the Mouse Double Minute 2 homolog (MDM2), which is itself regulated by p14/ARF. By binding to p14/ARF, MDM2 is inhibited and therefore p53 protein is active. p53 controls the expression of several genes responsible for cell cycle arrest, senescence, DNA repair and cell death. Mutations in the CDKN2A locus result in the deletion of p14/^ARF^, inducing the restoration of MDM2 activity and consequently the inactivation of p53 (Figure 1). The cell can therefore continue its progression through the cell cycle, without any checkpoint in case of genetic defect [16,19].

#### 2.1.2. Alterations in RAS/RAF/MEK Pathway

**Figure 2:**
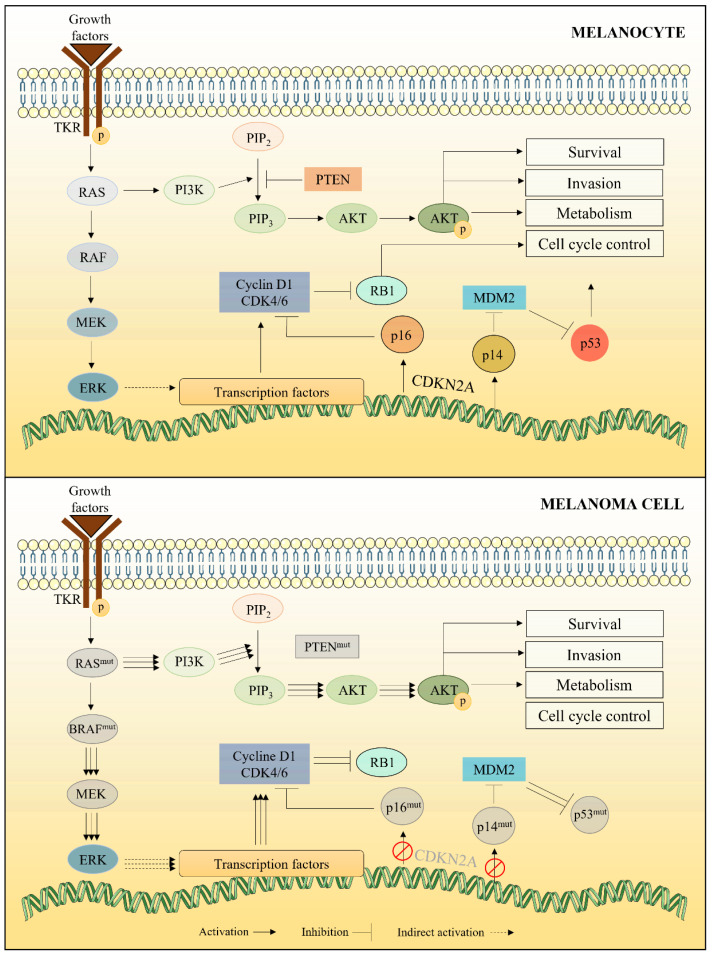
RAS/RAF/MEK signaling pathway in melanocytes, controlling cell cycle, cell proliferation and survival under normal conditions. In melanoma cells, mutations in RAS, BRAF, CDKN2A, p16, p14, p53 and PTEN cause constitutive activation of this pathway, resulting in excessive proliferative signaling.

Deregulation of the Mitogen-Activated Protein Kinase (MAPK) pathway, also known as the RAS/RAF/MEK pathway, is observed in many cancers, particularly in melanoma [20] (Figure 2). In healthy melanocytes, activation of this pathway begins with the interaction between extracellular growth factors and transmembrane tyrosine kinases receptor (TKRs). This interaction leads to the activation of RAS, a G protein with three isoforms (HRAS, KRAS and NRAS) responsible for triggering a cascade of reactions that lead to cell proliferation and survival. When stimulated, RAS forms a complex with one of the RAF isoforms (ARAF, BRAF or CRAF). The formation of this complex leads to the activation of RAF, which in turn phosphorylates and activates the MEK protein (MEK1 and MEK2 isoforms). This latter activates the MAPK isoforms (ERK1 and ERK2), which stimulate the expression of proteins that promote cell proliferation (cyclins, CDKs, etc.) and protect the cell from apoptosis by regulating the expression of pro- and anti-apoptotic proteins from the Bcl-2 family [21,22].

Overactivation of the RAS-RAF-MEK pathway is observed in about 90% of melanomas and the predominant mutation affects the BRAF gene in 50–70% of cases. 80% of BRAF mutations lead to the replacement of a valine by a glutamate unit at position 600 of the protein (BRAF V600E or BRAF^V600E^). This mutation makes the protein constitutively active, which generates proliferative stimulation even in the absence of growth factors [14]. NRAS, HRAS and KRAS isoforms are mutated in 15–20%, 2% and 2% of melanomas, respectively. The most common NRAS mutation (>80% of cases) consists of a replacement of a glutamine by leucine and arginine units at position 61 (NRAS^Q61L/R^) [16,21]. Similar to BRAF, mutation of RAS leads to constitutive activation of its GTPase activity.

#### 2.1.3. Alterations in PI3K/AKT Pathway

**Figure 3:**
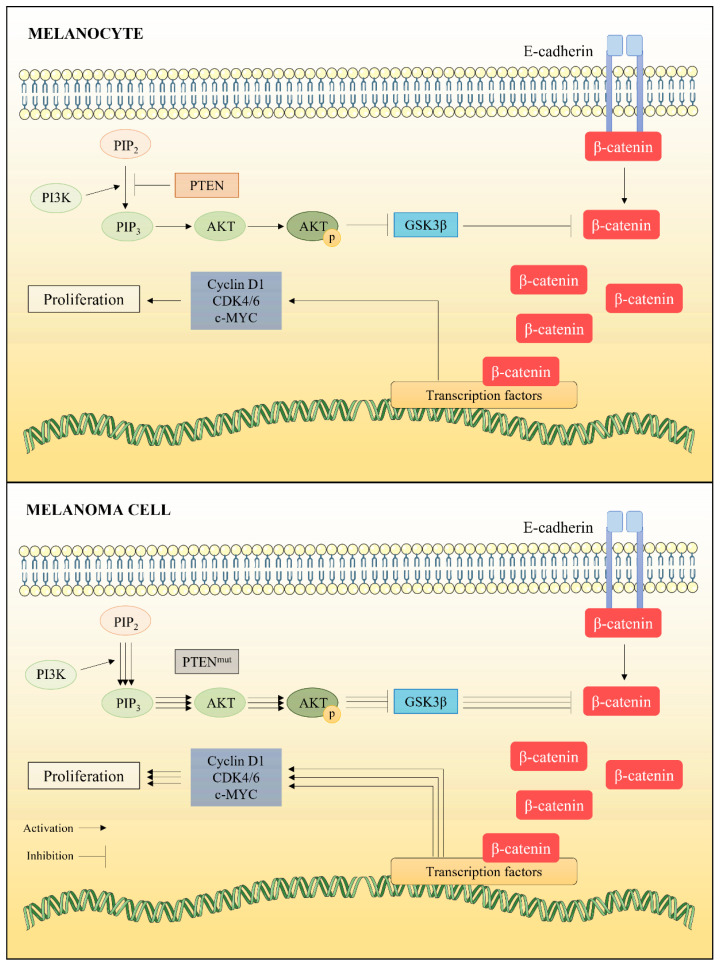
PI3K/AKT signaling pathway and its effects on β-catenin release and, consequently, cell proliferation. In melanoma cells, the main regulatory factor of this pathway (PTEN) can be mutated, promoting excessive cell proliferation.

Constitutive activation of RAS triggers not only overactivation of the RAF/MEK/ERK pathway, but also of the PI3K/AKT pathway, both contributing to the maintenance of proliferative signals. PI3K (phosphatidylinositol-3 kinase) is a member of the lipid kinase group, and its main function is the conversion of phosphatidylinositol-4,5-bisphosphate (PIP2) to phosphatidylinositol-3,4,5-triphosphate (PIP3). This latter activates the phosphoinositide-3-dependent protein kinase (PDK1), which is responsible for the phosphorylation of AKT (p-AKT) and, consequently, its activation [23]. Active p-AKT can phosphorylate several target proteins, including GSK3β, which is inhibited when phosphorylated. Due to this inhibition, free β-catenin can accumulate in the cell cytoplasm and move to the nucleus, where it can induce overexpression of important oncogenes, such as c-MYC and cyclin D1 (Figure 3). p-AKT thus prevents tumor cells from entering apoptosis and promotes cancer progression [24].

In melanocytes, PI3K/AKT pathway is controlled by PTEN (Phosphatase and Tensin homolog), an inhibitor of this pathway able to catalyze the dephosphorylation reaction of PIP3 to PIP2. Therefore, PTEN activity results in reduced levels of p-AKT, inhibiting proliferative cellular events induced by PI3K/AKT pathway [20]. However, the gene encoding PTEN is often altered in melanoma cells. This is the case in 7.3% of primary melanoma cells, 15.2% of metastatic melanoma cells and 27.6% of melanoma cell lines [25]. This mutation significantly reduces PTEN expression, allowing constitutive activation of the PI3K/AKT pathway.

### 2.2. Antimelanoma Therapy

Several options are available for the treatment of melanoma. The choice of the best therapeutic strategies depends mainly on two factors: disease stage and the patient’s clinical conditions (e.g., tolerance to treatment, liver, kidney or heart function, etc.) [26].

#### 2.2.1. Surgery

Surgery is the preferred treatment for non-metastatic stages of melanoma (I and II). Chemotherapy and/or radiotherapy may be combined with surgery to limit the risk of recurrence. Surgery consists of resection of the tumor area and possibly elective lymphadenectomy of regional lymph nodes to ensure that all cancer cells are removed [27]. A better understanding of tumor progression has led to refinements in the surgical procedure to ensure complete removal of tumor while limiting the size of the scar. This procedure generally removes all tumor cells, which explains the very low morbidity of melanoma in its early stages [28].

#### 2.2.2. Radiotherapy

Radiotherapy is based on irradiation of the tumor with high energy rays (rays γ, protons, hadrons). It is rarely used as first-line treatment for primary melanoma but may be indicated if the lesion cannot be surgically removed or when surgical margins are not perfectly established, leading to a high risk of metastasis or local recurrence. Radiation therapy can also be used as an adjuvant to surgical resection to ensure the complete elimination of cancer. However, it has many deleterious side effects for patients (e.g., burning, nausea, fatigue, hair and weight loss, neutropenia, etc.) [29,30].

#### 2.2.3. Conventional Chemotherapy

Chemotherapy for melanoma is based on the oral or intravenous administration of cytotoxic drugs, used in the more advanced stages (III and IV), when it has already acquired invasive and metastatic potential. The most used chemotherapeutic agents include DNA alkylating agents (e.g., dacarbazine, temozolomide, carmustine, cisplatin, carboplatin, etc.) and antimitotic compounds acting on microtubule polymerization, such as vinca alkaloids (vincristine and vinblastine) and paclitaxel [26].

Dacarbazine (DTIC) is one of the most widely used molecules to treat melanoma. It is a DNA alkylating agent that gives response rates of 10–20% after four to six months of treatment. However, clinical studies show that only 2% of patients receiving DTIC are still alive six years after treatment, and the combination with other drugs does not significantly improve survival rate [31].

Temozolomide, a prodrug that undergoes rapid conversion at physiological pH to monomethyl triazenoimidazole carboxamide (MTIC), a DNA alkylating agent with a structure close to DTIC, is indicated for brain metastases of melanoma but its efficacy is not significantly better than that of dacarbazine [32]. Similarly, vinca alkaloids and paclitaxel, respectively, polymerization inhibitors and microtubule stabilizers, yield response rates between 5 and 20% in the treatment of metastatic melanoma [33].

Combination of these cytotoxic agents increases the response rate to 20 to 30% but does not improve. In view of these low therapeutic efficiencies, combinations of cytotoxic agents with cytokines in so-called “bio-chemotherapy” protocols have been considered. In particular, the use of interferon alpha (IFN-α) and interleukin 2 (IL-2) associated with cytotoxic drugs has been evaluated for the treatment of metastatic melanoma [28,34].

#### 2.2.4. Bio-Chemotherapy

Combined treatment with dacarbazine and IL-2 and IFN-α has been considered since 1990s. There are several protocols associating both approaches with different doses [35]. IL-2 is a natural cytokine, secreted by T4 lymphocytes (LT4), whose role is to stimulate the proliferation and maturation of T lymphocytes and NK cells. Interferons (IFN) are secreted by monocytes and lymphocytes, especially T4 Th1, and act as a complement to IL-2 by increasing the phagocytic activity of macrophages and the cytotoxic activity of T8 and NK lymphocytes. Combination of these two cytokines induces strong immune response against tumor cells [36]. Bio-chemotherapy gives a higher response rate than chemotherapy alone (between 40 and 50% response in phase II clinical trials), but several studies conclude that it does not provide a significant increase in survival rate in metastatic melanoma. This treatment also has two major drawbacks: its high cost, which reduces its democratization, and its high toxicity, which limits its use to treat patients with preserved cardiac, pulmonary and renal functions [30].

Until 2010, alternatives for the treatment of metastatic melanoma were mainly limited to the use of dacarbazine, high-dose IL-2 and bio-chemotherapy, despite its toxicity. Overall, metastatic melanoma was difficult to treat, and 5-year survival rates were very limited. Starting in 2010, two promising treatment routes were developed: a new immunotherapy modality, based on the use of immunological checkpoint inhibitors, and targeted therapy [35]. At the same time, several research works have shown that the CAR-T strategy can be very effective in destroying metastatic melanoma cells [37].

#### 2.2.5. Immunotherapy by Blocking Inhibitory Lymphocyte Receptors

**Figure 4:**
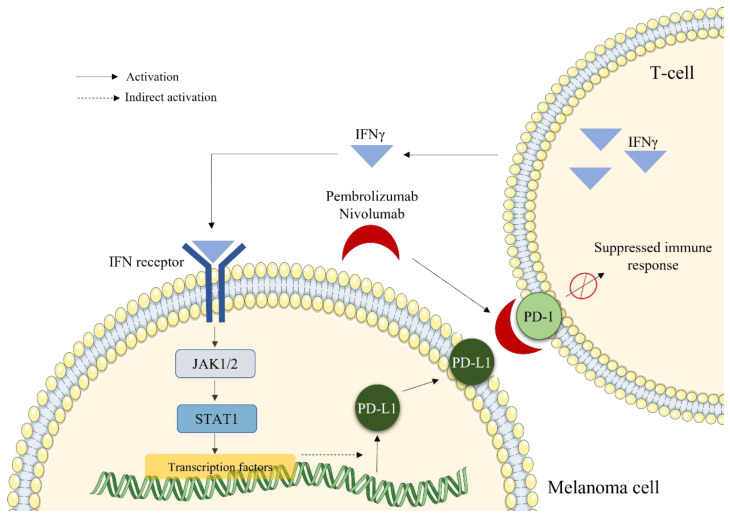
Mode of action of PD-1 inhibitors (pembrolizumab and nivolumab) used in immunotherapy for the treatment of metastatic melanoma.

A major advance in tumor treatment, awarded the 2018 Nobel Prize in Medicine to James Allison and Tasuku Honjo, was proposed in the 2000s [38,39,40]. This involves the use of inhibitory antibodies capable of specifically blocking two receptors that inhibit the anti-tumor immune response of T lymphocytes: PD-1 (Programmed cell death 1) and CTLA-4 (Cytotoxic T-Lymphocyte-Associated protein 4). PD-1 is a receptor expressed on T cells surface capable of suppressing the immune response when activated (Figure 4). Melanoma cells express PD-1 ligand (PD-L1) which induces T cell tolerance to tumor antigens and lack of cytotoxic response. Inhibition of the PD-1/PD-L1 interaction by antibodies or small molecules restores a balance in favor of a stimulatory co-activation signal that triggers T8 cytotoxicity on tumor cells. Monoclonal antibodies inhibiting the PD-1 receptor include pembrolizumab and nivolumab [41,42]. These drugs inhibit the PD-1/PD-L1 interaction and thus restore T8 antitumor cytotoxicity (Figure 4) [43].

Other human monoclonal antibodies (e.g., ipilimumab) have been developed to target CTLA-4 receptors, also expressed on T cells surface [44]. During the antigenic presentation process, upon interaction between the dendritic cell MHC class II and T4 cell TCR, a second membrane interaction between the dendritic cell factor B7 and the lymphocyte CD28 receptor is essential for T4 activation. This lymphocyte co-activation signal can be blocked by a specific inhibitory receptor, CTLA-4 (Cytotoxic T-lymphocyte-associated protein 4). Due to its higher affinity for B7, CTLA-4 anchors to this factor, preventing it from interacting with the lymphocyte CD28 receptor. Thus, a negative stimulus is triggered, resulting in T4 lymphocyte anergy and tolerance to the tumor antigen [45].

**Figure 5:**
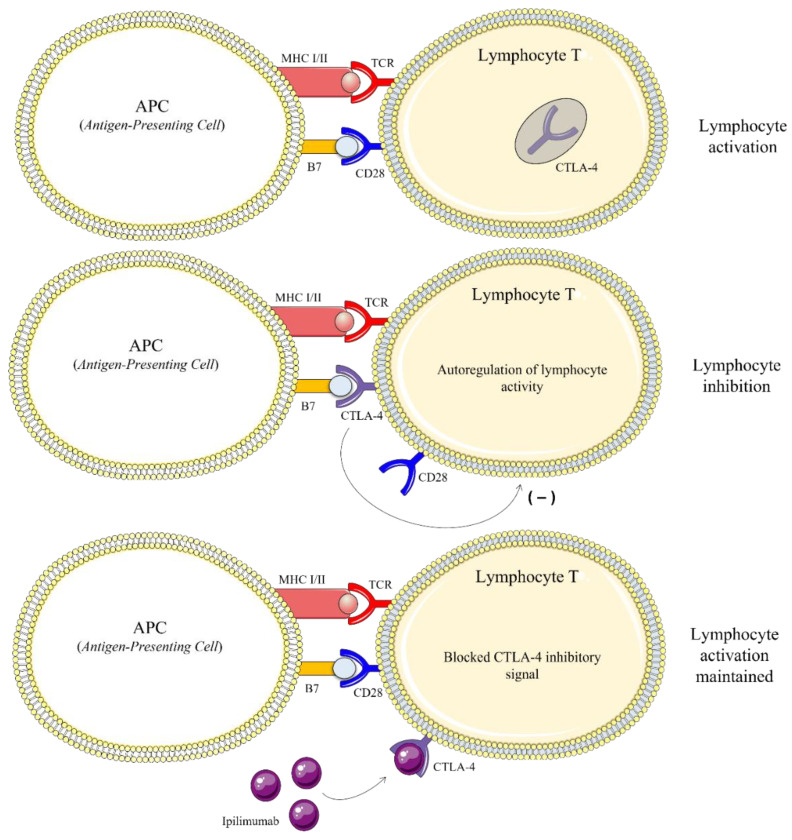
Mechanism of action of ipilimumab, used in melanoma immunotherapy. Ipilimumab blocks the CTLA-4 receptor that inhibits lymphocyte activity by interaction with factor B7.

By binding to CTLA-4, ipilimumab blocks the CTLA-4/B7 interaction and releases B7 to reactivate the lymphocyte via CD28 (Figure 5). The consequence of this stimulation is greater T-cell activity against cancer cells, but at the expense of an increased risk of immune events in other tissues. Recently, CTLA-4 and PD-1 inhibitors have become the main antibodies used in the treatment of metastatic melanoma (stages III and IV), whether or not combined with even more recently developed targeted therapy drugs [46].

#### 2.2.6. Targeted Therapy

One of the major advances in cancer treatment in past years has been the mapping of genetic mutations in tumors. This approach not only allows the identification of relevant pharmacological targets for each type of cancer, but also the achievement of a treatment adapted to patient’s genetic profile and to tumor progression stage. Key chemotherapeutic agents targeted for melanoma include inhibitors of the RAS-RAF-MEK pathway, particularly inhibitors of mutated BRAF (BRAF^V600E^) and MEK proteins [47].

Two BRAF inhibitors (BRAFi) targeting the BRAF^V600E^ mutation were approved in Europe and United States for the treatment of patients with advanced melanoma (stages III and IV): vemurafenib and dabrafenib [48,49]. A third molecule, encorafenib, is expected to be the next BRAFi to receive marketing approval [50]. These drugs are orally bioavailable small molecules that inhibit BRAF kinase. They have been shown to be more effective in the treatment of metastatic melanoma than conventional chemotherapy and bio-chemotherapy [47,51].

In addition to BRAF inhibitors, non-ATP dependent allosteric inhibitors of MEK1 and MEK2 kinases (MEKi) have been developed and introduced for the targeted treatment of metastatic melanoma. The main representatives of this therapeutic class are cobimetinib (MEK1 inhibitor), trametinib and binimetinib (MEK1 and MEK2 inhibitors). These drugs show better therapeutic efficacy than dacarbazine treatment in patients with BRAF, NRAS or both mutations [52,53,54].

MEKi and BRAFi have revolutionized melanoma targeted therapy, providing a solution to a therapeutic impasse. However, treatment with these drugs is often associated in the short term with acquired resistance mechanisms. About 50% of patients develop tumor progression after six months of treatment [47,55]. In contrast, preclinical and clinical studies have shown that combined therapy (BRAFi + MEKi) can limit resistance events and improve treatment efficacy [56].

**Figure 6:**
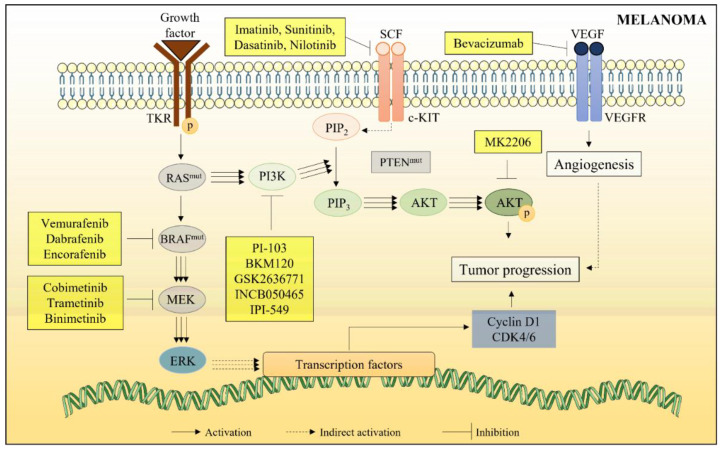
Main mechanisms involved in targeted therapy for metastatic melanoma.

Targeted therapy could also include inhibitors of key proteins involved in PI3K/AKT pathway, such as AKT inhibitors (e.g., MK2206), PI3K inhibitors (e.g., PI-103, BKM120, GSK2636771, INCB050465, and IPI-549), mTOR inhibitors (e.g., everolimus and temsirolimus), and c-KIT inhibitors, a receptor for the stem cell factor (SCF). VEGF receptor inhibitors (e.g., bevacizumab) have also been described as a possible alternative to control tumor progression by inhibiting the angiogenesis process (Figure 6). Due to their recent discovery, these chemotherapeutic agents are still in clinical development [26].

Despite the progress made with targeted therapy, these drugs still have a high toxicity due to the administration of high doses that limit their selectivity on tumor cells. Typical adverse effects are observed, including cutaneous, gastrointestinal, ocular, cardiac and musculoskeletal dysfunctions. Other side effects are specifically associated with certain molecules; for example, dabrafenib causes fever and vemurafenib induces photosensitivity in patients. Less common adverse effects may also be noted, such as anemia, facial paresis (encorafenib), neutropenia (dabrafenib), rash, and liver cytochrome induction (vemurafenib) [57]. Beyond its toxicity, the tumor response to targeted therapy is not sustained in a long term due to drug resistance mechanisms, which requires the use of escalating doses during treatment [58].

### 2.3. Mechanisms of Multidrug Resistance

A considerable number of patients show significant tumor progression within 12 months of starting treatment [59]. Chemoresistance mechanisms may pre-exist in tumor cells (particularly in melanoma, melanocytes themselves are cells with high resistance to chemicals due to their skin protection function) or emerge during treatment (acquired chemoresistance). Numerous alterations in signaling pathways explain acquired chemoresistance in melanoma [60], especially affecting RAS-RAF-MEK (MAPK) and PI3K/AKT pathways [61].

**Figure 7:**
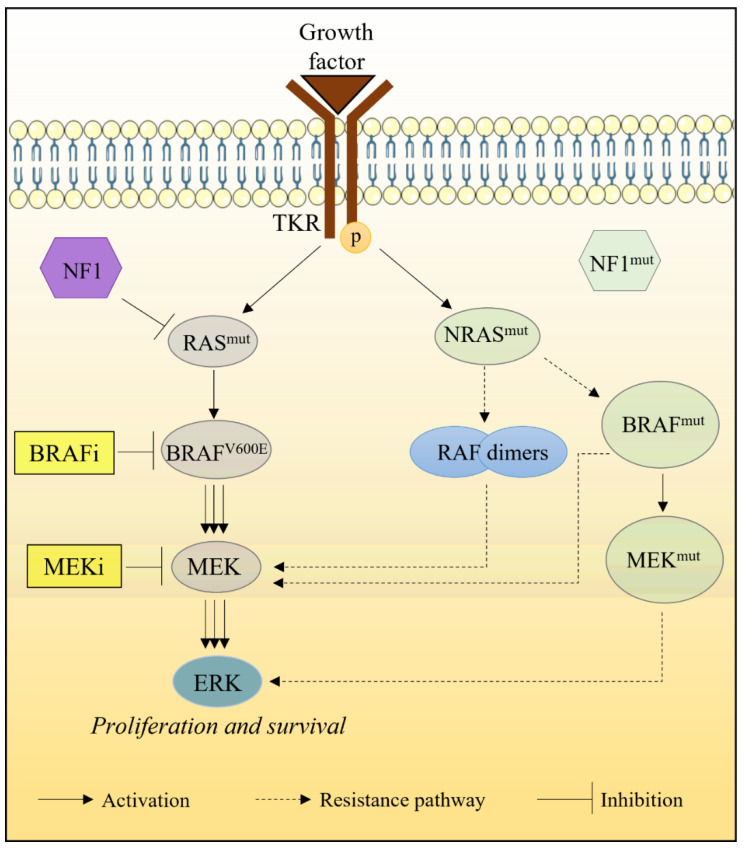
Aberrations in the MAPK (RAS-RAF-ERK) signaling pathway leading to chemoresistance. BRAF^V600E^ is inhibited by BRAFi, causing inactivation of the MAPK pathway. Amplification or alternative splicing of BRAF^V600E^ (BRAF^mut^) reactivates MAPK pathway in BRAFi-resistant tumors. In addition, the MAPK pathway can be reactivated by mutations affecting NF1, NRAS or MEK proteins in BRAFi-resistant tumors.

Absence of alterations in the RAS/RAF/MEK pathway is crucial for BRAFi clinical efficacy. Reactivation of this pathway in melanoma cells is therefore one of the main phenomena inducing acquired chemoresistance to BRAFi. Somatic mutations in the NRAS gene that affect residues G12, G13 or Q61 keep the protein in an active GTP-bound state, resulting in constitutive activation of the pathway. NRAS mutations are detected in approximately 20% of treated melanomas, particularly those treated with BRAFi [62,63,64]. Resistance to BRAFi can also be mediated by amplification (≅18%) or atypical splicing (≅14%) of BRAF^V600E^ [64]. Alternative splicing results in the expression of truncated BRAF^V600E^ proteins which do not contain the N-terminal RAS binding domain but preserve the kinase domain. The shortened BRAF^V600E^ proteins form homodimers that are resistant to BRAFi [65] (Figure 7).

Gain-of-function mutations have also been observed in genes that code for MEK1 and MEK2, making tumor cells resistant to MEKi. The major mutations associated with chemoresistance are MEK1K57N, MEK1Q56P, MEK1V60E, MEK1C121S, MEK1G128V, MEK1E203K, MEK2V35M, MEK2L46F, MEK2C125S and MEK2N126D [62,66]. The incidence of these mutations in tumors with acquired resistance ranges from 7–16% [62,64].

Mutations involving MAPK checkpoints have also been reported in chemoresistant melanoma. These include neurofibromin-1 (NF1), a protein that negatively regulates RAS activity by promoting the hydrolysis of GTP to GDP. In addition to loss-of-function mutations, NF1 activity can be decreased due to excessive proteasomal degradation [67]. Inactivation of NF1 increases HRAS, KRAS, and CRAF activities in mutated melanoma cells (BRAF^V600E^), restoring ERK activation even in the presence of BRAFi [68] (Figure 7).

**Figure 8:**
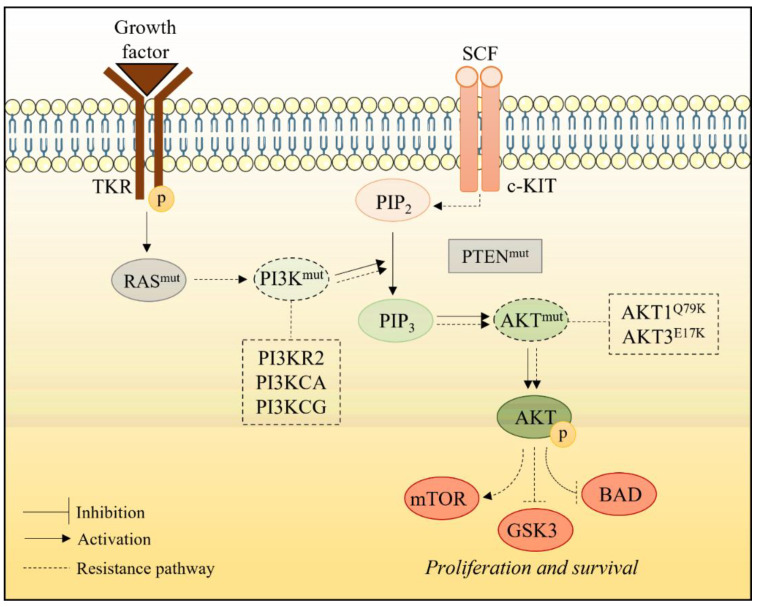
MAPK reactivation-independent mechanisms of resistance. Activation of RTK, mutations in PI3K or AKT, or loss-of-function mutations in PTEN, may mediate resistance to chemotherapy through reactivation of the PI3K/AKT pathway.

As in the RAS/RAF/MEK pathway, alterations in key proteins from PI3K/AKT pathway may occur in a treatment-dependent manner, leading to acquired resistance. Mutations in PI3K proteins (PI3KR2, PI3KCA and PI3KCG) have been identified in melanomas that have progressed after treatment with BRAFi. PI3K mutations increase AKT phosphorylation and decrease sensitivity to vemurafenib in vitro, suggesting these mutations may lead to BRAFi resistance in refractory tumors. Furthermore, this resistance phenomenon can be explained by activating mutations in AKT1 (AKT1Q79K) and AKT3 (AKT3E17K) [64], as well as by deletion of the major regulatory protein of this pathway (PTEN) during treatment [69] (Figure 8).

Given their impact on survival rates of patients with metastatic melanoma, multidrug resistance mechanisms have been exhaustibly investigated [58]. Any molecule capable of reversing chemoresistance or sensitizing tumor cells to chemotherapy or radiotherapy may indeed be of major interest for clinical oncology. Several research groups are dedicated to the study of new cytotoxic or cytostatic carotenoids with innovative modes of action. The objective is both to improve the antitumor response with the lowest possible dose of conventional drugs, limiting their toxic effects in non-tumor tissues and delay resistance mechanisms, and to consider alternatives to all-chemotherapy treatment, for example the development of nutritional interventions as adjuvant in antimelanoma therapy [70]. In this context, a renewed interest in microalgae carotenoids is perceptible, due to their structural chemodiversity suggesting the possibility of discovering original modes of action and of identifying molecules with little or no toxicity for chemosensitization, radiosensitization and/or reversion of multidrug resistance in metastatic melanoma.

## 3. Carotenoids from Marine Microalgae

Carotenoids are liposoluble pigments synthesized by terrestrial plants, micro and macroalgae, fungi, yeasts, bacteria and archaea. They are also found in animals, particularly crustaceans, fishes, birds, and mammals, where they are derived from the diet. In their producing or consuming organisms, carotenoids ensure numerous biological functions, such as photoreception (e.g., β-carotene, violaxanthin, zeaxanthin), membrane mechanical stabilization (thylakoids) (e.g., β-carotene), photoprotection and protection against oxidation (e.g., violaxanthin), as phytohormone and allelochemical (e.g., abscisic acid and 5-deoxystrigol), as well as visual, olfactory or gustatory chemoattractant (e.g., β-carotene, β-damascone, β-ionone), pro-vitamin A (e.g., β-carotene, β-carotene 5,6-epoxide) and transcription and cell differentiation factors (retinoic acid) [71].

To date (August 2022), 1204 natural carotenoids have been identified from 722 producing organisms. The Japanese Carotenoid Database (http://carotenoiddb.jp/ (accessed on 26 September 2022)) is the most comprehensive and detailed database to find updated information regarding structural diversity, biological functions, producing organisms, and classification of carotenoids. There are C30 (composed of 30 carbon atoms), C40, C45 and C50 carotenoids, distributed in all 3 kingdoms of life (eukaryotes, prokaryotes and archa ea).

**Figure 9:**
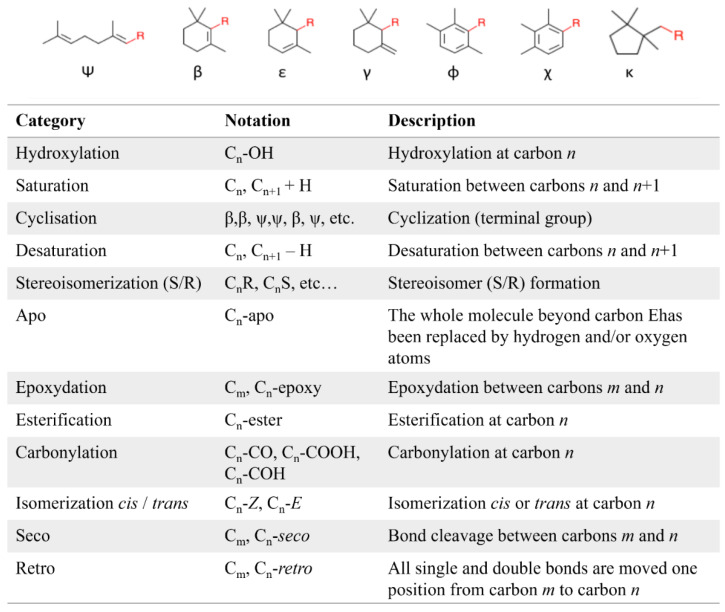
Illustration of the structural diversity of carotenoids. Possible terminal groups by cyclization and structural modifications identified in natural carotenoids. From http://carotenoiddb.jp/ (accessed on 26 September 2022).

Carotenoids whose chemical structure is composed only of carbon and hydrogen atoms are generally designated as *carotenes* and *lycopenes* while oxygenated carotenoids are grouped under the term *xanthophylls*. In fact, there is a high structural diversity of carotenoids, based on the possibilities of cyclization of terminal carbons, substitution pattern and chemical modification (hydroxylation, epoxidation, carboxylation, carbonylation, glycosylation, unsaturation, alkoxylation, isoprenic polymerization, lactonization, sulfation, cycloaddition, etc.) and isomerization (*cis/trans* isomerization) (Figure 9). Although they predominantly occur in *trans* configuration, carotenoids can be naturally obtained or converted to their *cis* configuration when exposed to heat, light, and acid media [72].

Although many carotenoids are described in plants, algae have been particularly employed in the development of carotenoid-enriched products as food supplements, cosmetics or even medicines. In this relatively recent scenario, microalgae have assumed an important role due to their easy cultivation, ability to adapt culture conditions on a large scale, possibility of production all year round, besides being considered a renewable source of carotenoids. Although the chemodiversity of microalgae is very high [73,74], it appears to be lower than that of macroalgae and especially terrestrial plants, which synthesize a wide variety of allelopathic molecules to resist predation. Some molecules with a completely original chemical structure are however found exclusively in microalgae, which underlines their potential for the discovery of original chemical motifs, which may present completely innovative pharmacological modes of action. In particular, carotenoids chemodiversity is very important and this family of molecules is the subject of much research in cancerology [75]. Indeed, they may be of major interest for the prevention, diagnosis, and treatment of cancers due to their numerous biological activities (antioxidant, photoprotective, antitumor, antimetastatic, antiangiogenic, photosensitizing, chemo- and radiosensitizing potential) [73,74,76,77].

**Figure 10:**
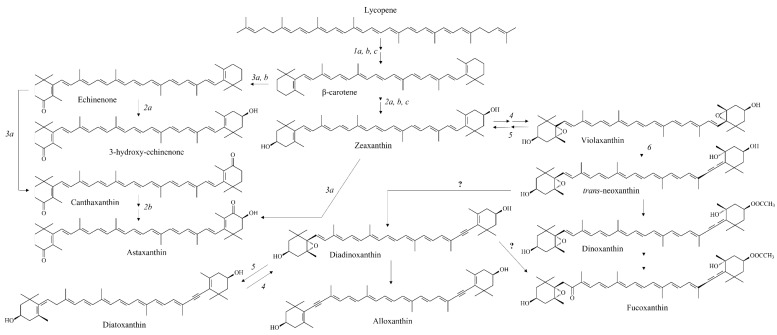
Structural diversity and major biosynthetic pathways of carotenoids found in microalgae. 1a, b, c: LCYB/CrtL-b, CruA and CruP (lycopene β-cyclase); 2a, b c: CrtR, CHYB/CrtZ, CYP97A (carotene β-hydroxylase); 3a, b: BKT/CrtW, CrtO (carotene β-ketolase); 4: ZEP (zeaxanthin epoxidase); 5: VDE (violaxanthin de-epoxidase); 6: NSY (neoxanthin synthase); ?: metabolic pathway unknown or not completely elucidated. Adapted from [3].

These liposoluble pigments, whether carotenes or xanthophylls, are present in all species of microalgae and play an important role in photosynthesis. Depending on the biosynthetic pathways present in each microalgal species, different chemical substitution patterns can be obtained, resulting in a large diversity of carotenoids. As in plants, carotenes are precursor molecules of xanthophylls. The metabolism of carotenoids starts from phytoene. A series of enzymes are involved in desaturation of phytoene to produce lycopene. Lycopene is converted into cyclic and then oxygenated carotenoids, with various functional groups (ketones, epoxides, hydroxyls, etc.). Figure 10 shows some examples of xanthophyll biosynthetic pathways in microalgae. Cyclases, epoxidases or de-epoxidases, ketolases, synthases and hydroxylases are among the main families of enzymes directly involved in xanthophyll biosynthesis. However, for some carotenoids the biosynthetic pathways are still unknown or not completely elucidated. This is the case for fucoxanthin, one of the most studied carotenoids in health [3], found in diatoms and brown algae. In addition, this was also the case for peridinine found in dinoflagellate, for alloxanthin which occurred in cryptophyceae, and for the couple diatoxanthin/diadinoxanthin common to diatoms and some dinoflagellates.

Certain unsaturation patterns are often found in microalgal carotenoids, such as acetylene (C≡C) and allene (C=C=C) systems observed in the hydrocarbon chain of alloxanthin and fucoxanthin, respectively. The acetylenic system, for example, is extremely rare in natural products. Acetylenic carotenoids are found exclusively in algae, with high abundance in microalgae [78]. Due to their structural originality, these molecules can exhibit pharmacological activities with a wide range of modes of action, especially in oncology.

## 4. Antimelanoma Potential of Carotenoids from Microalgae

**Figure 11:**
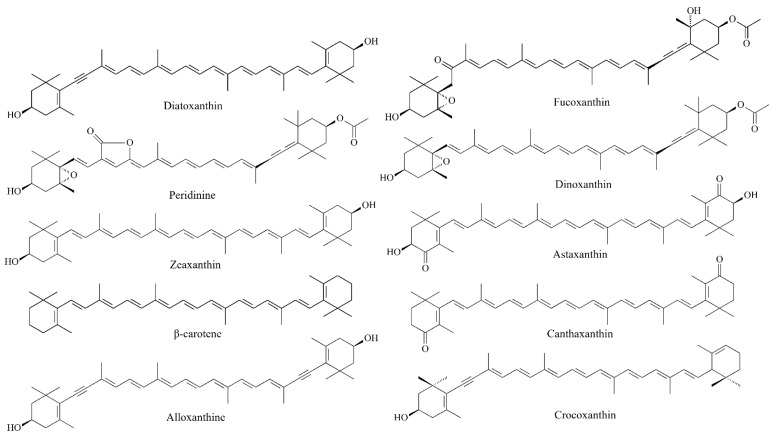
Examples of microalgal carotenoids showing anticancer activity in murine and/or human melanoma models.

Concerning melanoma, some carotenoids have shown significant cytotoxic, antiproliferative, pro-apoptotic and anticancer effects in in vitro and/or in vivo models. The leading carotenoids purified from microalgae and evaluated in murine and/or human melanoma models include: astaxanthin [79], fucoxanthin [7,10], canthaxanthin [80], zeaxanthin [9], β-carotene [81], crocoxanthin, alloxanthin [82], diatoxanthin, dinoxanthin and peridinin [83] (Figure 11). These molecules inhibit cell growth and drive melanoma cells to apoptosis by activating caspase pathway and inhibiting anti-apoptotic proteins from the Bcl-2 family. In addition, they can modulate pro-inflammatory signaling pathways involved in cell proliferation, such as the NF-κB pathway [9].

Some marine carotenoids induce cell cycle arrest through inhibition of CDKs (e.g., CDK4) and cyclins (e.g., cyclin D1 and cyclin D2), as well as inhibition of RB (p-RB) protein phosphorylation and increased expression of regulatory proteins p15 and p27 [7]. They also increase intracellular oxidative stress and cause suppression of metalloproteinases (e.g., MMP-1, MMP-2, and MMP-9), inhibiting metastatic cell migration [79]. Table 1 summarizes the main effects of carotenoids extracted from marine microalgae on melanoma cells. Most of the studies were performed in A2058 cells. A2058 are highly invasive and metastatic human melanoma cells, expressing the BRAF^V600E^ oncogenic mutation [84]. They are tumorigenic at 100% frequency in nude mice (supplier’s information) and resistant to conventional chemotherapy, especially alkylating agents such as dacarbazine. For this reason, this cell line is widely used not only to evaluate the antimelanoma potential of new compounds, but also to study their chemosensitizing potential when combined with conventional cytotoxic agents, such as BRAFi, MEKi and alkylating drugs. Considering their good clinical tolerability and acceptable safety profile, carotenoids have been increasingly envisaged in adjuvant therapy for the treatment of tumors. Although preliminary, studies focusing on melanoma are encouraging.

In general, carotenoids do not have a cytotoxic effect as potent as drugs used for the treatment of metastatic melanoma. However, when combined with chemotherapy, they are able to sensitize tumor cells and then reducing therapeutic doses of cytotoxic drugs [9]. One of the hypotheses put forward to explain their chemosensitizing effect is the integration of carotenoids into the cytoplasmic membranes of cancer cells, particularly at the lipid rafts, which can disrupt membrane fluidity, activate pro-apoptotic signaling pathways, and modulate the membrane transport of cytotoxic agents [6]. Next, we describe in detail investigations concerning key microalgal carotenoids with antimelanoma potential and possible molecular targets involved.

### 4.1. Fucoxanthin

Fucoxanthin is a xanthophyll abundantly occurring in brown seaweeds and contributes over 10% of the estimated total production of carotenoids in nature. Several pharmacological activities have been demonstrated for fucoxanthin, including antioxidant, anti-obesity, antidiabetic, anti-inflammatory and anticancer effects [88,89,90,91], which explains its use as a dietary supplement. Fucoxanthin induces apoptosis in a wide diversity of cancer cells and prevents in vivo tumor initiation, growth, angiogenesis and metastasis [90,92]. When combined with conventional anticancer drugs, fucoxanthin improves cytotoxicity against leukemia, colon, liver, breast and cervical tumor cells [93,94,95,96].

Previous studies have reported antimelanoma potential of fucoxanthin. Kim et al. [7] assessed its cytotoxic activity on B16F10 cells. Fucoxanthin-treated cells exhibited significant apoptotic body and nuclear condensation. After 24 h of exposure, fucoxanthin exhibited cell cycle arrest in G_0_/G_1_ phase, followed by a significative reduction of cells in S and G_2_/M phases. This effect was related to an important decreasing in the p-Rb level and an increasing in the p15^INK4B^ and p27^Kip1^ levels. Fucoxanthin treatment also promoted a concentration-dependent reduction in cyclins D1 and D2 and CDK4 levels. These checkpoint proteins play a critical role in regulation of the cell cycle and, consequently, in melanoma cells survival as shown in Figure 1 and Figure 2.

Fucoxanthin induced pro-apoptotic effect in melanoma cells by upregulating the expression of key apoptotic proteins such as caspases 3 and 9. Additionally, anti-apoptotic markers (BcL-xL, c-IAP-1, c-IAP-2 and XIAP) were found downregulated after fucoxanthin exposure, confirming its apoptosis-mediated cytotoxic activity [7]. The same study also investigated its antimelanoma potential in B16F10 cells-injected mice. Fucoxanthin-treated animals presented significant melanoma tumor mass reduction, confirming its antitumor effect.

A recent study using A2058 cells, expressing the BRAF oncogenic mutation (BRAF^V600E^), showed that fucoxanthin from the haptophyte *Tisochrysis lutea* is able to restore melanoma cells sensitivity to BRAFi (vemurafenib) and alkylating (dacarbazine) agents. Index combination (IC) values indicated fucoxanthin exhibits chemosensitizing activity by an addictive (at lower doses) or synergistic (at higher doses) behavior [10]. These outcomes suggest fucoxanthin is a promisor antimelanoma marine carotenoid with an encouraging potential to be use as adjuvant in melanoma treatment.

Concerning its safety profile, single-dose (1000 and 2000 mg/kg) and repeated-dose (500 and 1000 mg/kg, for 30 days) toxicity studies in mice demonstrate that fucoxanthin does not exhibit oral toxicity [97]. No death or histological abnormalities were observed in preclinical assays. In fact, fucoxanthin is widely used as a food supplement and its use as a pharmaceutical ingredient has become increasingly common, especially for the treatment of diabetes and obesity conditions [98].

However, we must consider that, in contrast with the extensive reports for other tumor cell types, the evidence for the use of fucoxanthin in the treatment of melanoma is still very preliminary. In addition, low stability and poor oral bioavailability are some limiting factors for the use of fucoxanthin, either as a main or adjunct treatment. Pharmacokinetic investigations point to a rapid metabolization of fucoxanthin after oral administration in mice, resulting in fucoxanthinol and amarouciaxanthin A available after one hour in the blood plasma. T_max_ of both metabolites was recorded at 4h after administration and C_max_ for fucoxanthinol was twice as high as amarouciaxanthin A. Fucoxanthin and both metabolites are predominantly accumulated in the liver, followed by lung, kidney, heart and spleen [99].

Stability studies also indicate that the pharmaceutical development of fucoxanthin-based products can be extremely challenging. Different studies have been conducted to evaluate the stability of fucoxanthin in its free form or in formulations such as emulsions, encapsulations and fucoxanthin-coated nanoparticles. Exposure to light, heavy metals, oxygen, high temperatures, enzymatic reactions, and long-term storage are among the main factors affecting the stability of fucoxanthin. Fucoxanthin is also sensitive to pH variation, and can be degraded under stomach and intestinal conditions, also leading to the formation of fucoxanthinol. In recent years, the use of emulsifiers and complexation with natural or modified polymers (e.g., cyclodextrins) has proven to be a promising strategy to preserve the stability of fucoxanthin in formulations [100,101,102].

Given the challenges in terms of pharmacokinetic properties, pharmaceutical development, and the preliminary nature of studies involving the use of fucoxanthin in melanoma, there is insufficient evidence to warrant the expected anti-melanoma effects in humans after oral administration. For this reason, we recommend further testing using in vitro and in vivo models, expanding to other cell lines before conducting clinical trials.

### 4.2. Astaxanthin

Astaxanthin is a xanthophyll carotenoid commonly found in plants, algae, and seafood such as fishes, shrimp and crab. Although it is mostly produced by macroalgae, microalgae (e.g., *Haematococcus pluvialis* and *Chlorella zofingiensis*) are also considered a great source of astaxanthin and have been recently used to provide high amounts to the development of dietary supplements [103]. In fact, due to its well-established safety and clinical tolerability, astaxanthin has been recognized by the United States Food and Drug Administration (FDA) and European Commission as a safe food color additive for food applications. Their uses are mostly based on its antioxidant activity and capacity to protect skin against UV-induced damage. Previous studies have also shown astaxanthin protects against inflammation, improves cell-cell communication and lipid metabolism [104].

Concerning its antimelanoma potential, astaxanthin was evaluated in two different cell lines: A375 and A2058 [79]. Preliminary data showed that astaxanthin has better cytotoxic effect against A2058 compared to A375 cells. After 24 h of treatment, astaxanthin reduced cell migration in both cell lines in a concentration-dependent manner. This finding was explained by an astaxanthin-induced decreasing on matrix metalloproteinase (MMP)-1, -2 and 9- expression, suggesting this microalgal carotenoid could prevent invasion and metastasis process. Astaxanthin induced sub-G1 cell cycle arrest and increased caspase-3 and -7 activity, showing an expected pro-apoptotic activity. All in vitro outcomes were confirmed in in vivo xenograft model. Treatments with astaxanthin (25 mg/kg daily, for 28 days) reduced tumor size by 76 and 82% compared to vehicle-treated group.

In a recent study, an oil-in-water (O/W) nanoemulsion loaded with astaxanthin was developed and tested in C57BL/6 mice bearing B16F10 melanoma tumor [85]. After oral treatment, astaxanthin nanoemulsion effectively triggered the apoptosis pathway, including enhancements of caspases-3 and -9 activity, ataxia-telangiectasia mutated kinase (ATM), and p21WAF1/CIP1 (p21). Bcl-2 expression was reduced as well as cyclins D1 and E, indicating astaxanthin controls important checkpoints of cell cycle. Astaxanthin was also able to inhibit MEK and ERK expression, commonly overexpressed in melanoma cells as shown in Figure 2. A significant anti-metastatic effect was observed in astaxanthin nanoemulsion-treated animals, accompanied by MMP-1 and -9 downregulation.

Although it has low oral toxicity and is widely used as a food supplement, astaxanthin is also considered a carotenoid with low oral bioavailability. The membrane permeability of free astaxanthin seems to be significantly affected by its low aqueous solubility and crystalline structure formation at body temperature. In general, naturally obtained or chemically synthesized astaxanthin esters provide an alternative to the use of astaxanthin in humans [105]. They are often more orally bioavailable than astaxanthin in lipid-based formulations and have better thermal stability [106]. Some astaxanthin ester derivatives (e.g., astaxanthin mono and diesters) obtained from green algae (e.g., *Haematococcus pluvialis*) even show improved antitumor effect against UV–7,12-dimethylbenz(a)anthracene (DMBA)-induced skin cancer model in rat [107].

### 4.3. Zeaxanthin

Zeaxanthin is an abundant carotenoid found in various dietary sources, including microalgae. A first report described the isolation of zeaxanthin from the glaucocystophyte *Cyanophora paradoxa* as one of the pigments responsible for its antiproliferative activity against the highly invasive human melanoma cell line A2058 [108]. A more recent study has evaluated possible mechanisms of action involved in its antimelanoma activity using this same cell line [9]. Zeaxanthin, this time purified from the rhodophyte *Porphyridium purpureum*, induced apoptosis-mediated cytotoxicity. Zeaxanthin-treated cells showed chromatin condensation, nuclear blebbing, sub-G1 phase cell cycle arrest, DNA internucleosomal fragmentation and activation of caspase-3. Western blot analysis revealed that zeaxanthin induced upregulation of the pro-apoptotic factors Bim and Bid and inhibition of NF-ĸB pathway. When combined with vemurafenib, zeaxanthin increased melanoma cells sensitivity, indicating its potential as dietary adjuvant in melanoma treatment.

Zeaxanthin also induced apoptosis in two human uveal melanoma cell lines (SP6.5 and C918) without impairing the cell viability of non-cancer uveal melanocytes [87]. Zeaxanthin-induced apoptosis was associated to downregulation of anti-apoptotic factors (Bcl-2 and Bcl-xL) and upregulation of pro-apoptotic markers (Bak and Bax). Zeaxanthin also evoked the release of mitochondrial cytochrome *c* and, consequently, caspase-9 and -3 activation. Its antimelanoma potential was validated in vivo in a nude mouse model, which tumor size was reduced by 56% in eyes treated with low dosages of zeaxanthin and 92% in eyes treatment with high doses after comparison with vehicle-treated animals [86].

### 4.4. Other Carotenoids

A bioprospecting investigation led to the purification of antimelanoma pigments from a non-toxic dinophyte, *Heterocapsa triquetra*, including carotenoids such as diatoxanthin, dinoxanthin and peridinin. After microwave-assisted extraction process, these carotenoids were tested on A2058 cells and exhibited low to moderate antiproliferative activity (25.6–34.5% of growth inhibition) [83]. Another preliminary investigation described the antimelanoma potential of canthaxanthin in SK-MEL-2 melanoma cells. After 48 h of exposure, cantaxanthin induced apoptosis in a concentration-dependent manner. A recent study reported the antiproliferative effect of alloxanthin and crocoxanthin on A2058 cells. These xanthophylls were extracted and purified from *Rhodomonas salina* and then its antimelanoma potential was assessed. Both carotenoids inhibited cell growth and migration, induced apoptosis and sub-G1 cells accumulation after 72 h of treatment. Alloxanthin potentiated the cytotoxic activity of vemurafenib (BRAFi) and restored melanoma cells sensitivity to dacarbazine (alkylating agent), contributing to a reduced resistance in A2058 cells.

## 5. Conclusions

Microalgal carotenoids have a remarkable structural diversity, which confers them a wide range of biological activities with diverse mechanisms of action. These compounds usually have well-established safety profiles and acceptable clinical tolerability. Many of them are used as food supplements. Concerning their antimelanoma potential, the xanthophylls purified from marine microalgae assume a leading role. They inhibit cell proliferation, migration and invasion, as well as induced cell cycle arrest and apoptosis. MAPK, NF-ĸB, MMP and apoptotic factors (caspases and Bcl-2 protein family) are frequently affected by microalgal carotenoids treatment. Fucoxanthin, astaxanthin and zeaxanthin seem to be the most investigated carotenoids in melanoma management. The antimelanoma potential of these three has been validated in in vivo experimental models. They not only exhibit direct antimelanoma effect but are also capable of restoring melanoma cells sensitivity to conventional chemotherapy (e.g., vemurafenib and dacarbazine). Although all preclinical data presented in this review are still preliminary, they have shown that it was possible to purify an important source of carotenoids from microalgae with interest to be used as adjuvant in melanoma therapy.

## Figures and Tables

**Table 1:** Main effects of microalgal carotenoids on melanoma cells. In order to better visualize the intervention possibilities of the marine microalgal carotenoids mentioned below, please check Figure 1, Figure 2 and Figure 3.

Carotenoid	Model	Cell Line	Dose (Route, Duration) or Concentration (IC_50_)	Main Effects and Molecular Targets Involved	Reference
Alloxanthin	In vitro	A2058	1–100 µM (IC_50_ = 29 µM)	Antiproliferative, inhibition of cell migration, pro-apoptotic activity (↑ caspase 3), chemosensitizing effect (combined treatment with vemurafenib)	[82]
Astaxanthin	In vitro and in vivo	A375, A2058	In vitro: 5–125 µg/mL (IC_50_ NR) In vivo: 25 mg/kg (*i.p.* daily, for 28 days)	Antiproliferative, inhibition of cell migration (↓ MMP-1, ↓ MMP-2, ↓ MMP-9), ↓ oxidative stress, cell cycle arrest (G1 phase), pro-apoptotic activity (↑ caspases 3 and 7)	[79]
	In vitro and in vivo	B16F10	In vivo: 10 mg (*p.o.* daily, for 35 days)	Antiproliferative activity, pro-apoptotic effect (↑ caspases 3 and 9, ↓ Bcl-2), ↓ cyclins D1 and E, ↓ MEK, ↑ p21, ↑ ATM, ↓ ERK, ↓ NF-ĸB, ↓ MMP-1, ↓ MMP-9, anti-metastatic activity	[85]
Canthaxanthin	In vitro	SK-MEL-2	1–10 µM (IC_50_ NR)	Antiproliferative and pro-apoptotic effect	[80]
Crocoxanthin	In vitro	A2058	1–100 µM (IC_50_ = 50 µM)	Antiproliferative and pro-apoptotic activity (↑ caspase 3)	[82]
Diatoxanthin	In vitro	A2058	100 µg/mL (IC_50_ NR)	Antiproliferative effect	[83]
Dinoxanthin	In vitro	A2058	100 µg/mL (IC_50_ NR)	Antiproliferative effect	[83]
Fucoxanthin	In vitro and in vivo	B16F10	In vitro: 12–200 µM (IC_50_ = NR) In vivo: 300 µg/100 µL (*i.p*. once every 5 days, for 20 days)	Antiproliferative, cell cycle arrest (G1/G0 phase), ↓ p-RB, ↓ cyclins D1 and D2, ↓ CDK4, ↑ p15, ↑ p27, pro-apoptotic activity (↑ caspases 3 and 9, ↓ BcL-xL, ↓ c-IAP-1, ↓ c-IAP-2, ↓ XIAP)	[7]
	In vitro	A2058	1–100 µM (IC_50_ = 14.67)	Antiproliferative activity, chemosensitizing effect (combined treatment with vemurafenib and dacarbazine)	[10]
Peridinin	In vitro	A2058	100 µg/mL (IC_50_ NR)	Antiproliferative effect	[83]
Zeaxanthin	In vitro	A2058	5–60 µM (IC_50_ = 40 µM)	Antiproliferative, pro-apoptotic effect (↑ caspase 3, ↑ Bim, ↑ Bid), ↓ NF-ĸB, cell cycle arrest, chemosensitizing effect (combined treatment with vemurafenib)	[9]
	In vivo	C918	114 µg and 570 µg (*i.o*., once)	Antitumor effect on human uveal melanoma model	[86]
	In vitro	C918, SP6.5	10–300 µM (IC_50_ = 28.7 and 40.8 µM, respectively)	Antiproliferative and pro-apoptotic effect (↓ BcL-xL, ↓ Bcl-2, ↑ Bak, ↑ Bax, ↑ caspases 3 and 9, ↑ cytosol cytochrome *c*), ↑ mitochondrial permeability	[87]
β-carotene	In vitro	B16F10	1–10 µg/mL (IC_50_ NR)	Antiproliferative, pro-apoptotic effect (↑ caspase 3, ↓ Bcl-2), ↑ p53, ↓ NO, ↓ iNOS, ↓ TNF-α	[81]

NO: nitric oxide. iNOS: inducible NO synthase. IAP: inhibitor of apoptosis protein. XIAP: X-linked inhibitor of apoptosis protein. MMP: matrix metalloproteinases. ATM: ataxia-telangiectasia mutated kinase. NF-ĸB: nuclear factor κ-light-chain-enhancer of activated B cells. ↑ indicates upregulation or increased activity. ↓ indicates downregulation or decreased activity. *i.p.* indicates intraperitoneal injection. *p.o.* indicates oral administration. *i.o.* indicates intraocular injection. NR: not reported.

## Data Availability

Not applicable.
